# Teaching Elaborative Reminiscing to Support Autobiographical Memory and Relationships in Residential and Community Aged Care Services

**DOI:** 10.3390/brainsci12030374

**Published:** 2022-03-11

**Authors:** Celia B. Harris, Penny Van Bergen, Paul A. Strutt, Gabrielle K. Picard, Sophia A. Harris, Ruth Brookman, Karn Nelson

**Affiliations:** 1 The MARCS Institute for Brain, Behaviour & Development, Western Sydney University, Penrith 2751, Australia; ruth.brookman@westernsydney.edu.au; 2 School of Education, University of Wollongong, Wollongong 2522, Australia; pennyvb@uow.edu.au; 3 Department of Psychological Sciences, Macquarie University, Sydney 2109, Australia; paul.strutt@mq.edu.au (P.A.S.); gabrielle.picard@mq.edu.au (G.K.P.); 4 Department of Psychiatry and Mental Health, University of New South Wales, Sydney 2051, Australia; sophia.harris@unsw.edu.au; 5 The Whiddon Group, Glenfield 2167, Australia; k.nelson@whiddon.com.au

**Keywords:** elaborative reminiscing style, conversational memory, ageing, aged care, person-centred care

## Abstract

Memories of the past are critically important as we age. For older adults receiving formal care in a range of settings, reminiscing with care staff may provide frequent opportunities for recalling autobiographical memories with a supportive conversational partner. Importantly, prior research suggests that some reminiscing conversations are more supportive than others. In the developmental literature, a long tradition of sociocultural memory research has shown how children’s autobiographical memory is scaffolded and supported by parents during reminiscing, when parents use a particular kind of conversational technique, known as “elaborative reminiscing”. In the current project, we aimed to examine whether we could enhance conversations between staff and older people receiving aged care by teaching care staff about these beneficial conversational techniques and supporting them to reminisce more often with residents/clients. We also aimed to determine whether staff members’ use of elaborative reminiscing techniques was associated with autobiographical memory details recalled by residents/clients during routine conversations. We conducted a workshop with 16 staff within a residential aged care and community care setting. We followed this with a 4-week training-and-feedback period during which staff recorded their conversations with residents and clients. Staff feedback indicated successful use of the scaffolding techniques overall, and benefits as well as barriers to their use in day-to-day practice. Analysis of the conversations demonstrated that the use of particular elaborative reminiscing techniques by staff was associated with increased recall of episodic and semantic autobiographical memory details by residents/clients. Overall, findings suggest that the principles of elaborative reminiscing may apply across the lifespan, and that the benefits of elaborative reminiscing for autobiographical memory may be particularly important in times of cognitive need. Practically, training aged care staff in specific and practical conversational tools can facilitate reminiscing for people receiving aged care.

## 1. Introduction

Sharing memories of the past in conversation with others is critically important across the lifespan, including as we age [1]. Remembering one’s own salient and everyday life experiences can support the on-going construction of personal identity, for example, with potential implications for wellbeing [2,3]. Moreover, the act of sharing memories with others offers opportunities for bonding, for eliciting emotion and sharing aspects of oneself, and for teaching others about life in the past [3,4,5]. For older adults receiving aged care, frequent day-to-day opportunities for reminiscing with care staff may therefore provide important cognitive and emotional benefits. Reminiscing together may offer a particularly rich form of social connection between older adults receiving care and their carers, as a way of enabling person-centred and relationship-based aged care [6]. While reminiscing offers numerous benefits across the lifespan, prior research suggests that some reminiscing conversations are more beneficial than others.

### 1.1. The Elaborative Reminiscing Style

Effective joint remembering depends on specific communication strategies that do not necessarily come naturally or intuitively to conversation partners. To understand the features of effective reminiscing conversations we turn to developmental psychology, noting the long tradition of sociocultural memory research showing how young children’s autobiographical memory is scaffolded and supported by parents during reminiscing [7,8,9,10,11,12,13]. As children take part in reminiscing conversations, parents use a range of conversational techniques to encourage and support their child’s burgeoning contributions [11,12]. The evolving memory narrative is therefore supported by one partner but co-constructed by both. These conversational techniques adopted by parents enable children to contribute more to the conversation than they otherwise would be capable of, and, with practice, to eventually internalise these same reminiscing skills themselves [14,15]. Research from the parent-child domain may have applications for older adults. Both younger children whose memory is developing and older adults who are experiencing cognitive decline or impairment may have difficulty with memory retrieval, and may also recall memories that are less detailed than those of their conversational partners [16,17]. Thus, both young children and older adults experiencing memory impairment may particularly benefit from a responsive and sensitive remembering partner who supports them to recall autobiographical memory details.

Importantly, there are strong individual differences in the extent to which parents provide effective memory scaffolding, and these differences may also apply to other lifespan contexts. According to the developmental literature, the use of memory scaffolding techniques sits on a continuum. At one end of the continuum, a “high-elaborative” style of scaffolding is characterised by the provision of event details and structure, open-ended questions that invite contributions, and evaluative feedback to motivate further participation [12,14]. A “low-elaborative” style, in contrast, may include scant detail or questioning and repetition of one’s own interests at the expense of the conversational partner. These differences between high and low elaborative styles have important impacts over time. Parents who are highly elaborative have children who recall more autobiographical memory detail themselves: first in the immediate shared conversation, as a direct consequence of the scaffolding, but also later during independent memory conversations with an experimenter [9,10,11]. Intervention studies have also shown that mothers can be coached to adopt a more elaborative reminiscing style [18,19,20], with immediate and long-term benefits for their children’s memory and development [18,21,22]. Given these findings, in the current project we examined whether it was also possible to teach conversational partners of older adults who had a cognitive impairment to use this high-elaborative reminiscing style.

### 1.2. Conversational Memory Scaffolding for Older Adults

The large body of theory and research on memory scaffolding and the elaborative reminiscing style has focused on conversations between parents and children. Far from being a developmental phenomenon, however, recent research suggests that memory scaffolding may be of lifelong importance. Harris and colleagues [23,24,25] compared individual and joint memory performance in long-married older couples, across a range of memory tasks including both word-list material and autobiographical material. They reported that at least some couples showed collaborative facilitation, whereby they recalled more when collaborating than when remembering separately, especially when the memory task was more relevant to the shared history of the couple. These collaborative benefits were associated with particular communication styles; namely cuing and prompting, sharing expertise, confirming and reiterating partner contributions, and avoiding corrections [25,26]. Thus, across two distinct contexts, at opposite ends of the lifespan, scaffolding from a loved one can support and extend the unfolding memory conversation with multifaceted benefits. Moreover, in both contexts, the degree of scaffolding experienced by the conversational partner (i.e., the child or older adult) is dependent on the presence of particular conversational and communication features. Given that similar links between conversational scaffolding and memory performance emerge for both younger children and older adult couples, we aimed to determine if such benefits would also emerge in contexts where older adults are likely to experience even greater need for memory support.

### 1.3. Conversational Scaffolding in Aged Care

Older adults receiving aged care or living within a residential care facility are at particular risk of cognitive decline and social isolation. Data from Australia highlight the high prevalence of cognitive impairment within aged care facilities, with a majority of people having a formal diagnosis of a cognitive impairment and an additional group who show evidence of a cognitive impairment without having been diagnosed [27]. Thus, this population may particularly benefit from the extra cognitive support that effective memory scaffolding provides. At the same time, people receiving aged care are often isolated from the social networks they may have had at other stages in their life, and are therefore at risk of loneliness [28]. Person-centred care from aged care staff may provide one key source of alleviating such loneliness, with research suggesting that aged care residents who require more hands-on care from staff have lower levels of loneliness, perhaps because of their higher levels of staff contact [29]. Moreover, with the recent lockdowns in aged care facilities associated with the COVID-19 pandemic, residents as well as older people living at home have been at increasing risk of loneliness and isolation. This is due to family members being unable to visit, and the cancellation of outings, activities, and community events [30]. For these reasons, aged care staff may play a key role in engaging aged care residents and clients in rich elaborative reminiscing conversations and thus, both staff and residents/clients may benefit from an intervention [31]. Importantly, conversations with staff might play an important role not just in alleviating loneliness but also in providing opportunities and support for residents/clients to recall autobiographical memories. Prior research has proposed that memory aids alongside carer training can enhance communication and conversations in aged care settings [32]. The elaborative reminiscing literature provides a well-established body of evidence from which to design such interventions for carer training.

### 1.4. Teaching Elaborative Reminiscing in Aged Care

In the current research, we aimed to examine whether principles of memory scaffolding and elaborative reminiscing could extend to improve conversations between older adults and care staff in an aged care setting. In doing so, we addressed two key questions. First, do the principles of elaborative reminiscing and individual differences in styles that have been observed with parents and long-married couples extend to non-familial scaffolding partners, such as care staff? Second, if care staff engage in elaborative reminiscing, are there demonstrable benefits for the older adults who are their conversational partners?

On the first question, we drew on emerging evidence of children reminiscing with their educators to highlight the role that non-familial reminiscing partners might play [33,34]. While educators are often not privy to children’s everyday experiences at home and may sometimes lack knowledge of children’s most salient life events, there is nonetheless consistent evidence of scaffolding for both shared and unshared autobiographical memories. Educators show individual differences in their use of an elaborative reminiscing style and different types of content with children, and children’s contributions to those same conversations are supported by their educators [33,35,36]. There are similarities between aged care staff and educators, as both professions include a caring role with frequent day-to-day interactions with a range of people in their care, and yet may lack the closeness and knowledge of shared history and experiences that family members can draw on for reminiscing.

On the second question, we drew on evidence for immediate and long-term benefits of the elaborative reminiscing style for children’s memory performance, contributions, and developing memory skills. Intervention studies identify the causal role that high-elaborative reminiscing plays in facilitating autobiographical memory recall [18,21,22], while longitudinal studies track differences in child outcomes into adolescence and early adulthood [37]. Notwithstanding the important differences between children and older adults, we considered the potential benefits of scaffolding for the memory performance of older adults receiving aged care services, such that the benefits of engaging in rich, supportive, elaborative reminiscing conversations in supporting autobiographical memory may be a lifespan phenomenon rather than a developmental one.

Importantly for the aged care setting, we considered the key role of responsivity in conversational scaffolding. For support from a conversational partner to be effective, it must enable the person who is being supported to perform at a level greater than they would be capable of independently, but not extend the person so far that they are unable to perform at that level even with support [38]. In the case of children, for example, parents may move from more frequent close-ended questions to more frequent open-ended questions as their children move into the preschool years and become more capable of verbally responding [12,15,22]. This notion of responsivity is particularly relevant for older adults with a cognitive impairment, who may be experiencing significant memory difficulties, and for whom even open-ended questions may be challenging. In such cases, an open-ended question that yields no response and is beyond the capabilities of the older adult might readily be followed by a close-ended question with greater conversational support. Older adults in aged care are a diverse cohort, with differences between individuals as well as within individuals whose cognitive capacity may fluctuate day to day [39]. In the aged care context, therefore, sensitive and attuned scaffolding means identifying what a conversational partner is capable of and extending their contribution slightly beyond what he or she can achieve themselves, but not further than he or she can achieve with support. In the current study, we examined different kinds of prompts, both open-ended and closed.

### 1.5. The Current Study

The aim of the current study was to implement an elaborative reminiscing intervention for care staff working in aged care. The study took place in an aged care residence and independent living village. The service is run across two Australian states by a not-for-profit provider with a network of homes and other aged care services, including community-based care. This provider already practiced an established model of relationship-based care, with care staff receiving annual professional development in how to implement care under these principles. Under this model, staff are encouraged to develop meaningful relationships with residents/clients, to get to know them as individuals, and to have one-on-one interactions. This strong foundation meant that any additional cognitive, emotional, or social benefits at the conclusion of our reminiscing program could be attributed to the implementation of memory talk specifically, and to the unique benefits that talking about the past might bring to older adults.

We introduced care staff participants to the elaborative reminiscing style in a practical and interactive professional development session. We particularly focused on the value of open-ended questions, following the resident’s/client’s lead, and centering the resident/client as an expert. We also discussed tips and tricks for initiating and extending memory conversations, including ideas for topics and prompts, use of physical photos and objects around the room, and personal disclosure to get conversation going and focus on relationship-building. Following the workshop, we tracked staff participants’ elaborative reminiscing with an older adult participant in their care.

This study was an exploratory pilot to determine whether elaborative reminiscing techniques are feasible and beneficial in an aged care setting. To determine how feasible and valuable the program was from the perspective of care staff, we collected rich qualitative data in surveys and interviews, examining not only their use of the elaborative techniques but also their perceptions of usefulness, barriers, and benefits. To examine the impact of elaborative reminiscing on residents’/clients’ autobiographical remembering, we scored recorded conversations for the extent to which care staff used the scaffolding techniques and the extent to which residents/clients recalled both episodic and semantic autobiographical memory details, and we tested for any associations between these two measures. To determine any impact of our reminiscing program on measures of wellbeing, we also obtained pre- and post- intervention measures of quality of life and loneliness from residents/clients, and of positive and negative aspects of caring from care staff.

## 2. Materials and Methods

### 2.1. Participants

This study was conducted with ethical approval from the Macquarie University Human Research Ethics Committee. We recruited sixteen dyads for this project (N = 32), with each dyad consisting of an aged care staff member paired with a resident or client. The aged care home and independent living village was located regionally in the state of New South Wales, Australia. Aged care staff included individuals working in residential care and community-based care who were invited to take part in a university research study focused on conversations and memory. Care staff participants were each paired with a resident/client participant who had given written consent to participate after discussing the project with their families. One resident withdrew from the project on the day of testing. Although residents/clients with a formal diagnosis of “no more than mild cognitive impairment” were invited to take part, several participants obtained Montreal Cognitive Assessment (MoCA) scores below 18 on the day of testing, indicating the possibility of a more significant cognitive impairment (see Section 2.2 below). In consultation with the institutional ethics board and the aged care organisation, we therefore sought additional proxy consent from the families of participants with scores below this cut-off. The process of pairing staff members and residents/clients was completed by the aged care organisation, pairing those who were already well known to each other. Characteristics of the participants are shown in Table 1 and Table 2.

### 2.2. Materials

#### 2.2.1. Care Staff

Dyadic Relationship Scale (DRS). The DRS [40] is an 11-item scale used to assess the caregiver’s perspective of the dyadic relationship strain associated with providing care. The scale is comprised of the positive interaction subscale (6 items) and the dyadic strain subscale (5 items), and individuals are presented with statements (such as ‘I learned good things about myself’ or ‘I felt resentful’ respectively) and asked to rate their agreement on a 1–4 point Likert scale (‘Strongly disagree’ = 1, ‘Disagree’ = 2, ‘Agree’ = 3, ‘Strongly agree’ = 4). Scores are summed such that higher scores on the subscales indicate greater positive interaction and greater dyadic strain, respectively.

Post-intervention Evaluation. Staff were asked to evaluate the conversation tools learned during the intervention and how they implemented them during practice, such as their effectiveness, ease of use, and number of residents/clients they practiced with. Staff were presented with questions (such as ‘To what extent have you put the conversational tools you learned into practice in your day to do interactions with residents/clients?’) and asked to rate them on a 1–10 Likert Scale rating specific to each question (e.g., ‘Not at all’ = 1, ‘sometimes’ = 5, ‘every time’ = 10). Staff were also presented with open-ended questions (such as ‘Have you learned anything new about a resident/client or had an experience that surprised you?’) to obtain qualitative insight into their experiences after the intervention.

#### 2.2.2. Residents and Clients

Audiometry Assessment. To assess whether participants had sufficient hearing to engage in conversations, pure tone audiometry assessments were conducted using Shoebox (SHOEBOX™ Audiometry, SHOEBOX Inc., Ottawa, ON, Canada). Residents/clients were asked to wear noise-cancelling headphones that delivered different tones at a range of frequencies, and to signal if they heard a tone. The researcher then marked the resident’s response on the Shoebox iPad, and a Pure Tone Average (PTA) value for both ears was obtained at the end of the assessment. Higher scores indicate greater sensorineural hearing loss. The ability to hear (in the better ear) at and below 25 dB is considered normal hearing range. Individuals who have difficulty hearing sounds between different dB ranges are categorised into the following hearing loss categories; mild (26–40 dB), moderate (41–55 dB), moderately severe (56–70 dB), severe (71–90 dB), and profound (>91 dB) (WHO, 1991).

Montreal Cognitive Assessment (MoCA). The MoCA [41] is a brief cognitive screening test designed to assist in the detection of mild cognitive impairment and dementia. The measure was designed to examine performance in five cognitive domains: attention and working memory, visuospatial abilities, language, learning and memory, and executive functioning. The measure has a total score out of 30. A score of 26 to 30 indicates normal cognition, 18 to 25 indicates mild cognitive impairment, 10 to 17 indicates moderate cognitive impairment, and less than 10 indicates severe cognitive impairment.

Autobiographical Memory. Residents and clients were asked to recall two autobiographical memories in detail: one that was a turning point in their life, and the other a recent event that occurred in the last 12 months. Memory prompts were taken from McAdams’ Life Story Interview [42], with the instructions simplified to reduce cognitive load on participants. Participants shared each memory for three minutes. If either or both initial memory prompts failed to elicit a memory, participants were then asked to think of a high point in their life, or a low point, or lastly to report any important memory from their life.

WHO Quality of Life Assessment (WHOQOL-BREF). The WHOQOL-BREF [43] is a 26-item self-report measure used to assess an individual’s quality of life. There are four measured domains: psychological (6 items), physical (7 items), environment (8 items), and social relationships (3 items). Participants are presented with statements which they are asked how often an event occurs (such as ‘how often do you have negative feelings such as blue mood, despair anxiety or depression?’), or how satisfied they are in relation to the statement (such as ‘how satisfied are you with your sleep?’), with each response rated on a 1-5 Likert Scale (e.g., ‘Very poor’ = 1, ‘Poor’ = 2, ‘Neither poor nor good’ = 3, ‘Good’ = 4, ‘Very good’ = 5). Items 3, 4 and 26 are reverse scored. Higher overall scores indicate better quality of life across each of the subscales.

DeJong Gierveld Loneliness Scale (DGLS). The DGLS [44] is an 11-item scale used to measure an individual’s experience of loneliness, with the possibility for refinement into a social loneliness subscale (5 items; related to deficiencies in their broader social network), and a emotional loneliness subscale (6 items; related to the presence of an intimate partner). Individuals are presented with statements such as ‘there are many people I can trust completely’ and asked to indicate their agreement on a 1-5 Likert Scale (e.g., ‘No!’ = 1, ‘No’ = 2, ‘More or less’ = 3, ‘Yes’ = 4, ‘Yes!’ = 5). The emotional loneliness score is obtained by summing the number of neutral and positive answers for those subscale items, whereas the social loneliness score is obtained by summing the number of neutral and negative responses for those subscale items. The total loneliness score is the sum of the two subscale scores, and higher scores indicate greater loneliness.

### 2.3. Procedure

#### 2.3.1. Pre-Intervention

Resident and client participants were introduced to two university researchers with expertise in conducting neuropsychological assessments. The researchers outlined the project and the assessment sessions. Verbal consent was obtained from each participant, in addition to written consent. One researcher administered the demographic questions, WHOQOL-BREF, DGLS and MoCA on an iPad, while the second researcher administered the Shoebox Audiometry Assessment and the autobiographical memory task. The order of these activities was counterbalanced, such that one participant would complete tasks with the first and then second researcher, and one would complete tasks with the second and then first researcher.

#### 2.3.2. Intervention and Practice

Care staff attended the workshop session in the context of their workday. Care staff initially provided signed informed consent before completing a pencil and paper version of the demographic survey and the DRS. Two researchers then conducted a 1-h workshop in which they outlined “10 techniques for having good memory conversations”. The principles of elaborative reminiscing, framed as “ten tips”, were discussed with care staff including specific examples of each technique, and suggestions for troubleshooting. The ten tips, based on the elaborative reminiscing literature as well as previous work with older couples, were: (1) use open-ended questions; (2) reduce close-ended questions; (3) use personalised cues from shared experiences and background knowledge; (4) echo/reiterate what resident says; (5) avoid corrections and focus on function; (6) acknowledge expertise; (7) use self-disclosure; (8) use props, such as photos and objects; (9) show engagement and listening; and (10) use everyday interactions as opportunities to reminisce. The format was interactive and discussion-based, with staff encouraged to share their own experiences about what kinds of conversational prompts were successful in engaging with their residents and clients.

At the end of the workshop, staff were asked to put the elaborative reminiscing techniques into practice over the following weeks, as they went about their day-to-day care tasks. Each staff member was given a “cheat sheet” that summarised the 10 techniques, as well as a small handheld audio recorder. They were asked to take audio recordings of themselves interacting with the resident/client they were paired with for the research project. They were encouraged not to aim for perfection when recording, but told that we were interested in what works and what does not work, such that examples of failure or of “work-in-progress” were just as useful to the project as examples of success. Staff were encouraged to practice the techniques with all residents/clients that they encountered during their care, but to only record interactions with the specific resident/client that they were paired with for the purposes of the research study. Due to the demands of obtaining a recording within an already demanding work context, and variations in staff rosters, we did not stipulate any further constraints on the context or manner in which the recording should take place. Therefore, as is necessarily the case in applied field research settings, recordings varied in duration, time of day, and success.

Two weeks after the workshop, the two researchers who had conducted the workshop visited the site and conducted informal “feedback interviews” with staff, with the goal of maintaining their engagement, reminding them to collect their audio recordings, and troubleshooting any challenges they were experiencing.

#### 2.3.3. Follow-Up Post-Intervention

Approximately one month after the intervention, two researchers returned to the site to repeat the WHOQOL-BREF, DGLS, and autobiographical memory task with resident/client participants. As before, each researcher obtained informed verbal consent from the participants he/she was working with before commencing the session. Because the session was shorter in length, all tasks were completed with the same researcher in a single session lasting approximately 30 min.

Researchers met with each care staff participant, collected their audio recorder, and gave them the post-intervention survey as well as the DRS. Both questionnaires were completed as pencil-and-paper versions on-site, during a brief 15-min session with each staff member in the course of their usual workday.

In the analysis below, we compared staff members’ pre- to post-intervention responses on the DRS, and resident/client pre- to post-responses on the WHO-QOL, DGLS, and autobiographical memory measures. Pre-intervention measures were obtained directly prior to the workshop, and post-measures were obtained one month later. We aimed to examine whether there was evidence of benefits outside the conversation itself. Given the small sample size, we used non-parametric Wilcoxon Signed Rank pair tests on each measure, comparing pre to post within-subjects.

#### 2.3.4. Coding and Scoring

We used NVivo 12 (QSR International) qualitative data analysis software to organise and analyse the interview data from staff feedback interviews. First, we transcribed interviews verbatim. Second, we used inductive content analysis to examine the transcripts for emergent themes relating to use of the conversational tools, opportunities for implementing them, and observed benefits and barriers or difficulties. We read each transcript multiple times, tagged each utterance relating to each of these themes, conducted independent coding checks to ensure agreement, and collated the final codes to examine how frequently different themes were mentioned and to understand staff experiences.

To analyse the staff practice conversations, we coded all utterances by the care staff member and the resident/client during the conversation. For care staff, we used deductive content analysis to score the extent to which they used the elaborative reminiscing techniques. We developed our own coding manual based on the 10 conversational techniques mentioned in our training workshop and coded each utterance for whether it represented one of these codes. We also noticed an abundance of utterances that we coded as ‘rapport-building’ or ‘banter’—these did not fit into one of our 10 elaborative reminiscing categories so they were counted separately.

To examine the autobiographical memory recall of residents/clients, we coded each utterance for whether it contained memory content. We used the well-established coding system developed by Levine and colleagues [16] to score the presence of “internal” (episodic) memory details and “external” (semantic) memory details, applying the coding manual formalised by Addis and colleagues [45]. Internal episodic details refer to a specific life event (e.g., “that was a lovely day”). External semantic details refer to more general events, or facts about one’s personal past (e.g., “Sometimes it gets windy on the harbour”).

## 3. Results

### 3.1. Did Aged Care Staff Use the Elaborative Reminiscing Techniques?

Our first research question asked whether staff were able to implement the elaborative reminiscing techniques in their day-to-day interactions with residents and clients, given that aged care was an entirely new context for this kind of intervention. We addressed this question by asking staff to report whether they had implemented the techniques, and to identify opportunities for, and barriers to, conversation. Data about uptake came from three sources: (1) the formal post-intervention questionnaire; (2) the informal feedback interviews we conducted with each staff member; and (3) the audio files that staff recorded of themselves interacting with residents/clients in the course of their day-to-day care duties.

#### 3.1.1. Frequency of Reminiscing

The post-intervention questionnaire alongside the staff interviews indicated that staff reported using the elaborative reminiscing techniques in their day care practice. Staff reported that implementing the techniques was relatively low in difficulty, and took “some” but not “a great deal” of extra time. Descriptives for ratings on the questionnaire are provided in Table 3, and formal qualitative coding of the staff interviews was used to understand and contextualise participant responses.

In terms of ratings of adoption, on the post-intervention questionnaire staff reported high rates of adoption and use of the elaborative techniques across most of the different residents and clients they interacted with during their work day (see Table 3). The qualitative feedback interview with staff yielded a more nuanced understanding of adoption and individual differences in the extent to which they were able to implement the elaborative reminiscing techniques. Some staff reported adopting the scaffolding techniques and finding them particularly useful, some reported that they already had been having these rich conversations with residents/clients, and some reported that engaging in such conversations was too demanding or time consuming. We consider these differences further in discussion of “barriers” below.

We asked staff to list the specific techniques they had been using the most, on the post-intervention questionnaire, via a free-text response box that allowed for multiple answers. The most common technique reported was using open-ended questions, used by the majority (9/16) of staff. During their feedback interviews, staff members reflected thoughtfully and deliberately about how they could use more open-ended questions in their interactions:

“You do kind of think how you’re going to ask not a yes or no question. With me, I’ve had to think, no, that’s gonna be a yes-or-no answer. So occasionally I’ve had to think about how I can rephrase it so that they have to answer with more than a yes or no.”

Other common techniques reported were using props, especially photo albums in the room (5/16). Staff also reported going back to participant intake records and using knowledge of residents/clients backgrounds to prompt more specific questions based on shared knowledge,

“Whenever we get someone new over there, I like to look through their case history first, and then just have a look what they really interested in and start that conversation like that until they can build up that trust with me.”

Moreover, 7/16 staff mentioned that the most frequently used technique was increased and deliberate engagement in reminiscing with the residents/clients, including a new focus on starting reminiscing conversations, active and responsive listening, and using opportunities to talk during routine care tasks.

#### 3.1.2. Scaffolding Techniques

Coding of staff practice conversations indicated that staff were able to implement the scaffolding techniques during routine care. Thirteen out of 16 staff submitted recordings of their conversations (ranging from 1–6 recordings per staff member). Across a total of 26 unique conversations recorded by staff members, we found evidence of the presence of many of the scaffolding techniques, as well as evidence of individual differences in their use (see Table 4). Our coding indicated that most staff utterances during conversations focused on rapport building and banter, and most questions were yes/no closed questions. However, staff were able to use approximately eight open-ended questions during an average conversation (see Table 4). Acknowledging and echoing were also frequently used to show engagement in the conversation. Other techniques, such as using self-disclosure, using shared event knowledge, and using props as reminders, occurred once or twice per conversation on average. Importantly, corrections were also very rare in conversations, consistent with our instruction that corrections should be avoided.

#### 3.1.3. Ease and Opportunities for Use

Staff reported that the conversational strategies were relatively straightforward, with low difficulty ratings on the post-intervention questionnaire (see Table 3). During the feedback interview, one staff member reported:

“And when you put it into practice? … it is a bit daunting, like 10 steps to have conversation, oh my god! But when you get in there, it just goes.”

Staff reported that the program added “some” but not “a great deal” of extra time into their workday (see Table 3). This additional time commitment should be considered. Perhaps because of the extra time that the program could take, staff mentioned finding new opportunities to have conversations during routine care: thus ensuring that elaborative reminiscing conversations did not necessarily require extra time, but could replace less meaningful interactions during regular activities and tasks. For instance, one staff member found that waiting for shower water to warm up provided a new opportunity to talk:

“[Now] it’s not just filling in time, while we’re waiting for the hot water to heat up. It’s, you know, bring something out of it.”

Two staff members talked about how focusing on reminiscing could make some care tasks easier. According to the first staff member, for example, reminiscing could put residents and clients at ease and reduce anxiety around personal care.

“I think it makes the other things a lot easier, really. Because you’re not just going to the toilet, you’re talking about, you know, their husband, and things that they used to do, or the pets that they owned, and just finding out a bit…. but you can see them light up telling someone a story.”

According to the second staff member, there were emotional and wellbeing benefits putting residents and clients at ease:

“If you just go in and have that conversation, then it makes for a more seamless day, because they’re less frustrated.”

Staff also reported that they learned new things about their residents and clients and that each successful conversation provided new information and insights which could be used to start future conversations. For instance, one staff member reported:

“So I found that quite useful. Like, just getting the information and then building each day and learning each day, instead of having… a long conversation, it helps out just building that.”

In this way, embedding reminiscing practice into everyday care settings can help to enhance relationships between staff and residents/clients, replacing less meaningful conversations with more meaningful ones:

“It does give a bit more of a meaningful conversation as well, instead of just the usual, ‘How’s your day?”

“I found before it used to be, you know, ‘How was lunch? How was your day? Anything on the TV?’ And then I think after this, it’s more like, oh, let’s see if there’s anything deeper we can talk about … gets them talking more than, ‘Today was okay’ or ‘I had fish for lunch.”

#### 3.1.4. Barriers and Difficulties

Despite the successes of the reminiscing program for some staff members, adopting the elaborative reminiscing techniques was not always straightforward. Although staff rated their adoption of the techniques highly on the formal questionnaire, the more open-ended interviews yielded a more nuanced picture, and many staff mentioned encountering specific barriers or difficulties. These included the demanding and time-intensive nature of their job, where they needed to prioritise basic care tasks:

“Like any job, we’re task orientated, we’ve got things to do, and we’ve got a timeframe to do them in.”

It also included specific aspects of their role and the contexts in which they had opportunities to have interactions with residents/clients. For instance, staff who had a more specific role, such as dispensing medications or running activities, had more difficulty finding opportunities to have a one-to-one conversation with residents/clients, as they were less likely to spend time with a particular individual. Some simply appeared to benefit more from the program than others, perhaps due to other individual differences in confidence, personality, or motivation.

Barriers also included specific features of residents/clients, including their degree of cognitive impairment, hearing impairment, or other individual differences that made it easier or more difficult to engage in conversations. As one staff member described these differences:

“Some days it’s very hard, because if they are just giving you ‘yes, no’, and you think, well I’ve exhausted nearly everything, what more can I say?”

One staff member reflected on the challenging adjustment period when residents enter aged care:

“So then they come in here, and they’ve lost everything… some of them aren’t ready to form relationships.”

Other staff members reflected on the communication barriers that come with their own background. For instance, staff who spoke English as a second language or who spoke in non-Australian accented English reported barriers to comprehension that made conversations more difficult.

Finally, some barriers identified were systemic, including issues about who was present at staff handovers and how information was shared between staff members to ensure all benefited from knowledge about residents/clients’ lives and preferences. Some of these barriers therefore could be addressed via organisational or systemic change, including changes to handover and knowledge sharing, and addressing staff shortages and workload.

Overall, across the staff questionnaire responses, their informal feedback provided to researchers, and their recordings of actual interactions with residents/clients, we found evidence that the staff were able to adopt the elaborative reminiscing techniques during their day-to-day care. Adopted techniques particularly included open-ended questions, as well as a more general focus on talking about the past and making an effort to have quality, engaged conversations. Although having reminiscing conversations did add extra work for staff, they were able to identify ways of embedding these conversations into regular practices, in the context of other care activities. However there were a range of barriers, and elaborative reminiscing was adopted by most, but not all, care staff during our program.

### 3.2. Were There Benefits of Elaborative Reminiscing in Aged Care?

#### 3.2.1. Carer Feedback

In terms of benefits, we asked staff to rate and report on improvements in their conversations. Staff members reported that their conversations with residents and clients had improved in length and quality. When asked to rate conversation length from 1–10, where 10 means much longer than before, they rated their conversations at 6.9. One staff member reported:

“The lady I’ve been speaking to normally, she’s just one-word answers. But now I’ve been getting more and more out of her. So yeah, it’s been exciting to learn a bit about her.”

Several staff members also reported noticing benefits in terms of resident mood and wellbeing, and how reminiscing could enhance the relationship between the carer and resident, as well as enable person-centred care.

“I’ve only ever seen him annoyed about something. But then I was in there and he was talking about his wife or something. And I said, ‘Where were you guys from?’ And then next second, he’s got out all of the photos he’s ever taken and showed me all of them … all this interesting stuff, and I just thought he was a grumpy old man.”

Finally, staff also noted reciprocal benefits of richer conversations, where they also experienced benefits of learning new things from the expertise of residents/clients.

“I learned a lot because I’m coming from another country. Everything is new to me. When you have a chat, I learned a lot about things here, what happened.”

#### 3.2.2. Benefits during Conversations

Across the 26 conversations captured by staff during their day-to-day care, we coded each for the presence of the scaffolding techniques in the utterances of the staff members, and for the memory content recalled by the resident or client, including both episodic and semantic autobiographical memory details. As noted above in Table 4, staff did include a range of the scaffolding techniques in their conversations, to a varying extent.

To examine the impact of these variances we first ran an exploratory factor analysis on the scores across the 10 scaffolding techniques in the 26 conversations, to examine how they clustered together. We dropped corrections, as there were so few with limited range (see Table 4). We used a varimax rotation and an eigen-value criterion of 1, and we used regression to give each conversation a score on each resulting factor. The resulting 4-factor solution explained 84% of the variance. Factor loadings are presented in Table 5. The scaffolding techniques clustered meaningfully into factors that we conceptualised as “prompting” (asking both open-ended and close-ended questions, as well as past-focus), “centering” (expressing engagement, showing attention, and positioning the resident as an expert), and “reciprocating”, (engaging in rapport building or banter, disclosing about oneself, mentioning shared experiences). Using props loaded by itself on the fourth factor (see Table 5).

We obtained correlations between staff members’ scores on these four factors during conversations and the number of episodic and semantic memory details recalled by residents/clients, as well as residents’/clients’ own measures of engagement, including rapport building and banter, acknowledgements, and echoing. When conversations had staff members high on prompting, residents/clients recalled more episodic memory details, *r*(26) = 0.450, *p* = 0.021, but not semantic details, *r*(26) = 0.258, *p* = 0.203. However, when conversations had staff members high on centering, residents/clients recalled both more episodic details, *r*(26) = 0.518, *p* = 0.007, and more semantic details, *r*(26) = 0.760, *p* < 0.001. When conversations had staff members high on reciprocating, there were weaker associations with episodic, *r*(26) = 0.321, *p* = 0.109, and semantic memory, *r*(26) = 0.398, *p* = 0.044, but a much stronger association with residents’/clients’ own rapport and relationship building statements, *r*(26) = 0.891, *p* < 0.001. Prop use referred to by staff was not associated with resident/client recall or rapport building. Overall, this analysis gave insights into the in-conversation benefits associated with the scaffolding techniques, with their use associated with differential impacts on episodic memory, semantic memory, and relationship building.

#### 3.2.3. Pre- to Post-Intervention Measures

For staff responses on the ‘positive interactions’ subscale of the DRS, the Wilcoxon Signed Rank pair test was significant, W = 2.16, *p* = 0.031, indicating a general tendency for scores to increase pre- to post-intervention. Nine participants showed an increase, four remained the same, and two decreased. For staff responses on the dyadic strain subscale of the DRS, the Wilcoxon Signed Rank pair test was not significant, W = −1.07, *p* = 0.283. Nine participants showed a decrease, one remained the same, and five showed an increase. Overall, these results suggested an increase in positive interactions reported by staff from pre- to post-intervention.

For the resident/client responses across measures, there were no significant changes from pre- to post-intervention. This included the WHO-QOL, *W* = −1.05, *p* = 0.293, the social subscale of the DGLS, *W* = 1.73, *p* = 0.084, the emotional subscale of the DGLS, *W* = 0.79, *p* = 0.429, and the number of words spoken during the autobiographical memory task, *W* = −0.94, *p* = 0.347. Overall, from the residents’/clients’ data, we did not find evidence of general improvement in wellbeing or independent memory performance over the four weeks of the intervention.

## 4. Discussion

In the current research we aimed to translate principles of elaborative reminiscing into an aged care setting, building on research demonstrating the scaffolding of autobiographical memory that can occur during conversations between parents and children, educators and children, and members of older couples, as well as intervention studies suggesting that elaborative reminiscing can be taught [11,22,26,33]. We delivered a workshop about the principles of elaborative reminiscing to aged care staff in aged care residential and community care settings. We asked staff to record themselves practising these techniques with residents/clients in their day-to-day care, as well as to give us informal and formal feedback about their experiences. We measured whether staff member’s use of these techniques during conversation was associated with the extent to which residents/clients recalled episodic and semantic autobiographical details in response. We also gave staff a measure of positive and negative aspects of caregiving, pre- and post-intervention, and we measured residents’/clients’ quality of life, loneliness, and autobiographical memory recall.

Our results indicated that staff members found the principles of elaborative reminiscing useful and applicable to their practice within the aged care setting. They reported use of the techniques during both their formal and informal feedback, and were also observed to be using the techniques in their recorded conversations. Most importantly, we found evidence that use of the elaborative reminiscing techniques during conversation was associated with the extent to which residents/clients recalled autobiographical details. This novel finding extends the elaborative reminiscing literature, grounded in a long tradition of developmental research on parent-child conversations, and applies it in an entirely new context of aged care. In this way, we argue that principles of memory scaffolding are lifelong, rather than being specifically developmental processes.

### 4.1. Different Types of Scaffolding Techniques

We selected the 10 specific scaffolding techniques by drawing together the elaborative reminiscing literature [11,12,37,46] as well as previous findings on the benefits of specific communication techniques in the joint memory performance of older couples [25,26]. These literatures demonstrate striking commonalities in the communication techniques identified as beneficial, including cuing and asking questions, sharing expertise and following the partner’s lead, and showing engagement through acknowledgements and mirroring. Interestingly, in our principal components analysis, we found that the instances of the 10 techniques within conversations clustered together in meaningful ways.

#### 4.1.1. Prompting

Open-ended questions and close-ended questions both loaded together, alongside questions specifically about the past, in a factor we labelled “prompting”. This cluster contrasted somewhat with the developmental domain in which open-ended questions vs. close-ended questions are considered at opposite ends of a dimension of elaborative reminiscing. In the aged care context, particularly when reminiscing with people who have varying degrees of cognitive impairment, sensitive scaffolding may include both open-ended and close-ended questions, with close-ended questions functioning to get a conversation started, or to provide an even stronger scaffold if an individual is having difficulty remembering. Similarly, in prior research with older couples, both successful and unsuccessful cuing attempts loaded together on a beneficial, group-focused factor [25,26]. Together, these findings suggest that the focus on the past, and the attempt to prompt the other partner during conversational reminiscing is most important, regardless of success. Both open-ended and close-ended questions can serve this role in conversations between aged care staff and residents/clients.

#### 4.1.2. Centering

Statements that positioned the resident/client as expert, as well as echoing and acknowledgements, loaded together in a factor we conceptualised as “centering”. The instances of utterances associated with centering had the strongest positive relationship with both episodic and semantic memory details recalled during the conversation by residents/clients. This finding is consistent with prior research, demonstrating that statements about uneven expertise in older couples were associated with poor joint recall [25], as well as that older people particularly value opportunities to share their memories with others for the purposes of teaching and informing younger generations [3]. In the developmental domain, centering has parallels with following the child’s lead to elaborate on what they say, prioritise what is important to them, and focus on meaning-making during high-elaborative reminiscing [47]. In terms of aged care practice, centering aged care residents and clients as experts and partners in their care can facilitate person-centred care and enhance well-being [48]. We propose that centering statements on the part of care staff change the dynamics of care, creating a social environment in which residents and clients are motivated to recall and share memories.

#### 4.1.3. Reciprocating

Using self-disclosure, referencing shared events, and utterances related to small talk and banter loaded together on a factor that we conceptualised as “reciprocating”. Self-disclosure and talking about shared events were both techniques that we had encouraged care staff to use in order to make conversations more genuine, and small-talk/banter was an extra code we added when we observed the high frequency of these kinds of statements in recorded conversations. These strategies were less directly motivated by prior literature, and instead were aimed at providing strategies to compensate for the lack of shared history that we had anticipated may impact on the quality of conversations between staff and residents/clients. We considered that reciprocating would be important for creating a natural social context for reminiscing. Rapport- and relationship-building statements were the most common utterance made by staff during conversations, consistent with the warm, intimate, person-centred and relationship-based care they had already been trained to practice.

### 4.2. Benefits for Autobiographical Memory and Relationships

Importantly, we identified benefits for residents/clients associated with the use of elaborative reminiscing techniques by care staff. In the recordings, we found that different techniques were associated with different outcomes. In terms of associations with increased memory content, both “prompting” and “centering” statements by staff members were associated with increased episodic and/or semantic memory detail recalled by residents/clients. Thus, very specific elaborative reminiscing behaviours were associated with increased recall of autobiographical memory details. Prompting—asking open-ended questions—is the hallmark of the elaborative reminiscing technique because it invites a response. Prompting was particularly associated with recall of specific episodic memory details, suggesting that a targeted question about a particular event can support older adults to recall a specific event. Centering—making the resident/client the expert and following their lead—was also associated with both episodic and semantic information. The impact of centering on semantic recall suggests that these kinds of conversational techniques particularly enabled residents/clients to share their knowledge and expertise, consistent with the importance older adults place on using their autobiographical memories to teach younger generations [3]. In contrast, reciprocating statements were only associated with residents’/clients’ rapport-building small-talk, indicating that these kinds of statements do contribute to conversation and invite participation from residents/clients, but these statements were not associated with increased recall of autobiographical memory details.

This distinction aligns with staff members’ self-report. A number of staff members reported that they felt they already were engaged in excellent conversations with residents/clients and therefore did not particularly benefit from the workshop. Our analysis of the conversations indicated that staff did engage in a great deal of rapport-building small talk, and that such utterances were successful in engaging residents/clients who reciprocated with rapport-building small talk themselves. While such utterances may be beneficial for reciprocity and relationship building, and may enhance well-being and provide enjoyment to both conversational partners, they were not associated with supporting the recall of autobiographical memory content specifically. Thus, the nuanced distinction between having conversations that are enjoyable and support relationship building versus using the specific techniques that that prompt and scaffold memory is important.

The benefits that we identified for both scaffolding autobiographical memory details and for broader relationship-building are consistent with the benefits identified in parent-child conversations, where elaborative reminiscing has both cognitive and socio-emotional impacts that are evident during the conversations themselves, but also last long-term [21,47,49]. We only examined immediate impacts in the current project, particularly within the conversation itself. We did measure residents’/clients’ mood, wellbeing, and independent autobiographical memory recall over the course of the program but did not find any significant change. It is therefore possible that in the context of reminiscing where one person has a cognitive impairment, the benefits for memory and wellbeing are identifiable only during the conversation itself, and only in the presence of the scaffolding partner. However, our 4-week period was likely too brief to observe an impact, particularly with a small sample that had high levels of between-subject and within-subject variability. Longer term research with a larger sample size is warranted to determine whether engaging with care staff who adopt the high elaborative reminiscing style has measureable on-going impacts on long-term trajectories of ageing and adapting to aged care, including both cognitive and socio-emotional outcomes, in addition to the benefits we observed within the conversations themselves.

### 4.3. Barriers to Elaborative Reminsiscing

Notwithstanding the positive benefits of elaborative reminiscing for relationship building and for prompting residents’/clients’ memories, we also identified a range of barriers to the implementation of elaborative reminiscing, which warrant further investigation. First, we identified that there were individual differences in uptake, and not all staff reported successfully implementing the reminiscing techniques. In this small pilot, it was not possible to examine the role of individual difference factors, such as gender, personality, education, or job satisfaction in influencing the extent to which staff adopted elaborative reminiscing techniques. Specific barriers identified by staff included workload and role, particularly where staff endorsed a view of psychosocial needs as separate from or secondary to physical care [50]. Other staff found that reminiscing could be done in the context of physical care, and even facilitated such tasks on occasion, endorsing a more integrated approach to psychosocial and physical care [50]. Future interventions could more directly and comprehensively address these barriers during training and practice phases, leveraging the examples we have identified in the current project of creative ways to integrate reminiscing into the day-to-day routine.

### 4.4. Limitations and Future Directions

This project represents a promising first step in establishing the applicability of elaborative reminiscing techniques into aged care settings, in which staff found opportunities to have conversations about memory with residents and clients, many of whom had some degree of cognitive impairment. It was a small pilot with a variety of limitations, although it yielded rich data and insights. Future research will focus on replication with a larger sample, providing further opportunities to examine individual differences in both staff uptake of the elaborative style and resident/client benefits following this uptake. One limitation of the current data is that we did not collect a baseline of conversations prior to the intervention. Thus we do not have evidence that our intervention increased elaborative reminiscing behaviours, and our results may reflect natural variations in tendencies to be elaborative, similar to those identified in mothers [11]. Future research will aim to establish a baseline of conversational styles and their impact prior to intervention so that the effects of intervention can be measured against this existing baseline. Most of our care staff and resident/client participants were women, reflecting workforce and aged care demographics more generally. Future research could examine any influence of gender, and whether men and women differ in the kinds of conversations that are beneficial for them, given reliable gender differences in autobiographical reminiscing [51]. Due to the practicalities of implementing this program within a busy day-to-day aged care practice, there were variations in the duration, time of day, and the context of recordings obtained by staff, and the impact of these factors can only be examined with larger scale research. Finally, future research will seek to extend the intervention and analysis to family members of older people with a cognitive impairment, to examine whether they also vary naturally in their tendencies to elaborative reminiscing and whether they can benefit from learning about elaborative reminiscing techniques.

## 5. Conclusions

Our research study is the first to extend the well-established phenomenon of elaborative reminiscing out of the developmental context and apply it to the aged care setting. We found that learning about the techniques of elaborative reminiscing was useful for aged care staff, such that they adopted a focus on reminiscing and particularly the use of open-ended questions. Staff reported benefits for their relationship with residents/clients and for their care practices. We also identified benefits for the residents/clients within the conversations themselves, such that use of the elaborative reminiscing techniques on the part of care staff was associated with more memory details recalled by residents/clients. Thus, we propose that memory scaffolding is not specifically a developmental process in childhood, but a lifelong activity that has particular resonance in times of cognitive need. Our research represents the first attempt to formalise an elaborative reminiscing intervention for aged care staff, enabling them to provide a cognitively and socially supportive environment within the context of their day-to-day care.

## Figures and Tables

**Table 1:** Characteristics of care staff participants (n = 16).

	Mean (SD)	Distribution or Range
Gender	n/a	15 women, 1 man
Care setting	n/a	12 residential, 4 community
Employment type	n/a	14 part-time or casual, 2 full-time
Age (years)	43.27 (19.91)	24–60
Education (years)	12.93 (1.28)	10–14
Experience (years)	10.13 (7.43)	4–28

**Table 2:** Characteristics of resident/client participants (n = 15).

	Mean (SD)	Distribution or Range
Gender	n/a	11 women, 4 men
Care setting	n/a	10 residential, 5 community
Marital status	n/a	7 married, 6 widowed, 2 single
Age (years)	85.07 (5.27)	77–94
Education (years)	10.8 (2.21)	6–15
MoCA	15.60 (6.47)	6–28
Hearing (dB, better ear)	36.13 (9.90)	20–56.25

**Table 3:** Post-intervention questionnaire ratings by staff members.

Variable		Mean (SD)	Range
Extent of use	Extent of use (1–10)	8.19 (1.42)	5–10
	Range of use (1–10)	7.73 (1.33)	5–10
Difficulty and opportunity	Difficulty of use (1–10)	2.88 (1.74)	1–7
	Extra time (1–10)	4.69 (2.12)	1–8
Benefits	Length of conversations (1–10)	6.69 (1.44)	5–9
	Change in conversations (Y or N)	11/16 (Y)	n/a

SD = standard deviation; Y = Yes, N = No.

**Table 4:** Number of instances of scaffolding techniques during practice conversations.

	Mean (SD)	Range
Open-ended questions	7.96 (6.56)	1–24
Yes/no questions	17.31 (17.50)	0–74
Past focus	10.73 (13.18)	0–50
Acknowledgements	17.23 (18.36)	0–56
Rapport	18.69 (14.89)	3–69
Using event knowledge	1.73 (2.27)	0–8
Echoing	9.92 (12.12)	0–41
Corrections	0.12 (0.33)	0–1
Expertise	6.54 (7.49)	0–28
Self-disclosure	2.50 (4.84)	0–22
Using props	1.96 (2.38)	0–8

**Table 5:** Factor loadings for the elaborative reminiscing techniques.

	Component			
	Prompting	Centering	Reciprocating	Props
Open-ended questions	0.845	0.154	0.224	−0.133
Yes/no questions	0.810	0.391	0.232	0.186
Questions about the past	0.941	0.193	0.052	0.141
Acknowledgements	0.092	0.882	0.346	0.061
Echoing	0.243	0.914	0.146	−0.040
Resident as expert	0.425	0.804	0.094	0.130
Rapport/small talk	0.177	0.111	0.870	0.203
Shared knowledge	0.421	0.393	0.640	0.035
Self disclosure	0.068	0.198	0.924	−0.068
Props	0.071	0.059	0.085	0.977

Shading illustrates variables that loaded together across the 4 factors.

## Data Availability

Data are held by the first author and anonymised data files may be obtained upon request.
